# Too Little, Too Much: Evidence for Quadratic Patterns in Emotion Regulation Strategy Use in Daily Life

**DOI:** 10.1007/s42761-026-00390-9

**Published:** 2026-07-20

**Authors:** Dominique F. Maciejewski, Yasemin Erbaş, Tak Tsun Lo, Egon Dejonckheere, Merlijn Olthof, Andrea Bunge, Eeske van Roekel

**Affiliations:** 1 Tilburg School of Social and Behavioral Sciences, Tilburg University, Warandelaan 2, Tilburg, AB 5037 Netherlands; 2 Tilburg Experience Sampling Center, Tilburg University, Warandelaan 2, Tilburg, AB 5037 Netherlands; 3 Behavioral Science Institute, Radboud University, Houtlaan 4, Nijmegen, XZ 6525 Netherlands; 4 Faculty of Behavioural and Social Sciences, University of Groningen, Broerstraat 5, Groningen, CP 9712 Netherlands

**Keywords:** Emotion regulation, Dynamics, Daily diary, Emotion characteristics

## Abstract

Emotion characteristics, like emotion intensity and perceived controllability, shape how individuals regulate their emotions. While the link between emotion characteristics and emotion regulation strategies seems clear for some strategies, findings are mixed for others. This study proposes that individuals are most likely to engage in emotion regulation at average levels of emotion intensity and controllability, and less likely at low or high levels (i.e., when emotions are either too weak to require regulation or too overwhelming to regulate). To test this hypothesis, we used data from a 60-day diary study in 79 adults (mean age = 22.29, *SD* = 6.08, 84% female), who every evening reported on emotion characteristics (emotion intensity and controllability) and strategy use (distraction, rumination, reappraisal, expression, suppression, relaxation). In addition to single strategies, we also examined emotion regulation strategy switching (i.e., the extent to which individuals varied their strategy composition). We replicate some linear findings, including that expression, relaxation, and rumination were used on days with more intense and uncontrollable emotions (for expression only). Importantly, we provide novel evidence for quadratic relations, where suppression and reappraisal were used on days where emotions were of average intensity and controllability, and less on days where emotions were less or more intense and controllable than usual. Moreover, individuals were more likely to switch to strategies they typically do not use on days where emotions were less or more controllable than usual. These findings highlight the value of moving beyond linear relations when studying emotion regulation dynamics in daily life.

**Supplementary Information:**

The online version contains supplementary material available at https://doi.org/10.1007/s42761-026-00390-9.

Emotion regulation, the attempt to change emotions using strategies like distraction or suppression, is shaped by emotion characteristics (Aldao, 2013; Kalokerinos & Koval, 2024). Two key emotion characteristics are emotion *intensity* (how strongly individuals experience emotions) and emotion *controllability* (how much control individuals think they have over emotions). Earlier studies have shown that negative emotion intensity is related to increased emotion regulation strategy use, including more rumination (De France & Hollenstein, 2022; Lennarz et al., 2019; Medland et al., 2020), expression (De France & Hollenstein, 2022; Medland et al., 2020), and relaxation (De France & Hollenstein, 2022; Medland et al., 2020), possibly because intense negative emotions disrupt daily life and prompt regulation (Barrett et al., 2001). Findings for other strategies are more mixed. While some studies link higher emotion intensity to more suppression (Lennarz et al., 2019; Medland et al., 2020; O’Toole et al., 2017) and distraction (Lennarz et al., 2019; Medland et al., 2020; O’Toole et al., 2017; Petrova et al., 2024), others find no associations (Blanke et al., 2022; De France & Hollenstein, 2019). Reappraisal shows especially inconsistent results: Some studies find positive associations between emotion intensity and reappraisal (Hiekkaranta et al., 2021; Medland et al., 2020), others negative (Blanke et al., 2022; Petrova et al., 2024), yet others find no association (De France & Hollenstein, 2022; Lennarz et al., 2019; O’Toole et al., 2017).

In addition to intensity, perceived controllability plays a role in emotion regulation by influencing someone’s motivation and confidence to regulate. When individuals perceive emotions as uncontrollable, they may experience stress and reduced motivation to regulate, as such emotions may seem resistant to change (Ford & Gross, 2019; Hong & Kangas, 2022). However, while questionnaire and experimental research support this notion (Ford & Gross, 2019), empirical evidence in daily life is mixed or inconclusive: Limited research suggests that higher controllability is related to lower rumination and higher reappraisal, although no relations have been found with distraction, suppression, relaxation, or expression (Medland et al., 2020; Petrova et al., 2024).

We propose that some of the inconsistent and non-significant findings are because previous studies have typically assumed linear associations. Linear models implicitly assume monotonic changes in regulation effort (e.g., that regulation simply increases when emotions become more intense). This, however, may obscure patterns where individuals disengage from regulation at extreme levels of intensity and controllability. The central hypothesis of this paper is that quadratic (in particular, inverted u-shaped) relations may better capture how emotion characteristics are related to strategy use. Specifically, in line with recent Experience Sampling Method (ESM) studies on reasons that people do not engage in regulation (e.g., Daniel et al., 2024; Lai et al., 2026; Uchida et al., 2026), we argue that both very low and very high intense emotions are less likely to be regulated. When emotions are not intense and individuals feel they are very controllable, individuals may not feel the need to regulate, because overall there is little to regulate (e.g., Lai et al., 2026). This may especially be the case in diary studies, that often involve quite mundane situations (Kuppens et al., 2022). In contrast, when emotions become too intense or feel too uncontrollable, individuals may fail to regulate, because they become overwhelmed and have little confidence in themselves in regulating (e.g., Daniel et al., 2024).

While most prior research has focused on the relation between emotion characteristics and *single* strategies, recent work suggests that individuals dynamically vary their use of *multiple* strategies to meet contextual demands (Aldao et al., 2015; Blanke et al., 2020; English & Eldesouky, 2020)). One important aspect of these multi-strategy dynamics is strategy switching (Aldao et al., 2015; Lo et al., 2024). Strategy switching refers to changing from using one strategy to using another strategy, rather than increasing or decreasing the effort devoted to a single strategy (or to the same set of strategies). For instance, an individual may for most of the time engage in reappraisal to deal with negative emotions, but on one day switch to distraction, for instance when reappraisal is deemed ineffective or the context changed. A recent study has shown that momentary strategy switching predicted lower negative emotion intensity hours later (Lo et al., 2024). However, how emotion characteristics shape daily strategy switching remains poorly understood.

## The Present Study

Using data from a 60-day diary study, we examined the within-person associations between emotion characteristics (intensity and controllability of negative emotions) and related emotion regulation strategy use in daily life. We examined six strategies targeting different emotion components: cognition (distraction, rumination, reappraisal), behaviors (expression, suppression), and physiological arousal (relaxation; De France and Hollenstein (2017)). We hypothesized *linear* within-person relations between emotion intensity and strategy use (Hypothesis 1; positive direction) and between emotion controllability and strategy use (Hypothesis 2; non-directional due to limited within-person research). We also tested for *quadratic* within-person relations. We hypothesized an inverted U-shape relation, where emotion regulation strategies would be used less with emotions of low intensity/controllability and high intensity/controllability (Hypothesis 3; directional), but due to limited research did not hypothesize for which strategies quadratic effects would be most likely. We also conducted two exploratory analyses. First, we tested whether emotion intensity/controllability was related to emotion regulation variability. Second, based on recent research that has found that individuals did not regulate intense negative emotions especially when they perceived them as uncontrollable (Petrova et al., 2024), we tested whether emotion intensity and controllability interacted in predicting strategy use.^1^

## Method

### Transparency and Openness

This manuscript uses the Workflow for Open Reproducible Code in Science (WORCS version 0.1.14, Van Lissa et al., 2020) to ensure reproducibility and transparency. In this paper, we report how we determined our sample size, all data exclusions, all manipulations, and measures in the study. The study’s hypotheses and analysis plan have been pre-registered (https://osf.io/fg58t/?view_only=7711b0c0057346f58d10192a324ae817). The analysis code is available at https://osf.io/f42un/?view_only=ba769f827a04424389311f66db8fdeb1 and the data can be downloaded at the university’s data repository after signing a Data Use Agreement (https://doi.org/10.34973/s90j-0r08).

Data for this paper had already been collected. The justification for the number of subjects and time-points are detailed in the project description (Maciejewski et al., 2022) and included designs of similar studies and resource constraints (see also Lakens, 2022). We also conducted an a-priori power analysis using Monte Carlo Simulations with 1000 replications following procedures from Lafit et al. (2021) using parameter estimates from another pre-existing dataset (Medland et al., 2020). That study is comparable in many aspects, including the sample (university students) and instrument (same items), but differ on sampling scheme: Our study sampled once a day over 60 days and assessed only the most intense negative emotion during that day, whereas the study by Medland et al. (2020) sampled ten times per day for seven days and assessed the most intense negative emotion in the last hour. We purposefully focused on the single most intense negative emotional event each day. First, we believe that this instruction decreases ambiguity and improves interpretability for participants, as it ensures that they report on regulation strategies that are tied to a clearly defined emotional experience rather than a diffuse or ambiguous set of emotions. Additionally, this instruction likely captures greater variation in emotion characteristics and reduces floor effects in negative emotions, a common limitation in ESM studies with frequent intra-day measurements (Maciejewski et al., 2023). For instance, ESM studies indicate that individuals report not engaging in regulation in more than 50% of the time (Daniel et al., 2024; Lai et al., 2026; Livingstone & Isaacowitz, 2021). Thus, while our study likely suffers from more recall bias, it probably has a stronger signal-to-noise ratio. For most, but not all, combinations between emotion characteristics and emotion regulation strategy use, there was sufficient power (> .80) for either the linear or the quadratic effect (i.e., there was only sufficient power for the linear, but not the quadratic effect or the other way around; see Supplementary Material 2). We nevertheless decided to include all models in our study, because the power analyses were not intended to guide a decision about model inclusion, but interpretation of results (i.e., results for the potentially underpowered model combinations should be treated in an exploratory way). More details can be found in the pre-registration (https://osf.io/fg58t/?view_only=7711b0c0057346f58d10192a324ae817) and in Supplementary Material 2.

### Participants and Preprocessing

Participants came from the *Track your Mood* project. In total, 83 participants started the study and 6 participants dropped out over the course of the study. As outlined in our pre-registration, observations were excluded if they indicated possible technical errors, including observations with a negative time between sending and opening the questionnaire, which did not occur, and if the questionnaire was opened after it should have expired ($n_{obs}$ = 29). Additionally, observations were excluded if the participants completed the 28-item questionnaire in less than 1 minute ($n_{obs}$ = 125), because such short response time likely indicate careless responding (Jaso et al., 2022). Lastly, participants were excluded if they showed zero variance across time variation in study variables, because this could also indicate careless responding (*n =* 4). Further inspecting those participants showed that these concerned participants with only 1 or 2 observations in total. In total, 160 observations (5.09%) and 4 participants were excluded. The final sample consisted of 79 participants and 2983 observations. The mean age was 22.29 (*SD =* 6.08, range = 18 - 53) and 88.61% were students, of which 84% identified as women, 11% as men, 1% as non-binary (no information was available for 4% of the sample).

### Procedure

The *Track your Mood* project was approved by the ethical Committee of the Faculty of Social Sciences, Radboud University Nijmegen (Protocol number: ECSW-2021-075). The project had two broad aims, namely 1) studying the associations between emotions, events, and emotion regulation strategy use in daily life and 2) studying the effect of micro-interventions on momentary affect. Participants were recruited through convenience sampling (i.e., via advertisements at the university) and through the university’s online participant registry. Data collection took place from October 2021 to December 2021 in the Netherlands.

During an onboarding session (either in person or online), participants signed the informed consent, after which they automatically received an e-mail to fill in a baseline questionnaire via Qualtrics (e.g., about mental health, personality). Those questionnaires are not relevant for the present research questions and are described elsewhere (Maciejewski et al., 2022). Together with the research assistant, participants downloaded the *m-Path* app (Mestdagh et al., 2023)) which was used for the ESM data collection. The research assistant discussed the ESM questionnaire with the participants to make sure that they understood the items (e.g., by asking the participants to give examples of the items). The ESM period began the same day and lasted 61 days in total (onboarding day and 60 days of ESM assessment). Due to a mistake, 18 participants only received notifications for 60 days (including onboarding day). Participants received five notifications per day with a fixed signal-contingent sampling scheme. The first four notifications were scheduled at 9:00, 12:00, 15:00 and 18:00 hours and contained one bipolar momentary emotion item (“Right now I feel...” with a scale from very bad [0] to very good [100]). The fifth notification was scheduled at 21:00 hours and contained a bipolar momentary emotion item and questions about emotions, events, and emotion regulation strategies (in total 28 items; see below). The present study only uses data from the evening questionnaire. Each questionnaire was available for two hours. Participants could adjust the exact times to fit their personal schedule during the onboarding session.

After study completion, participants could either receive a gift voucher (VVV or bol.com coupon) for 25 Euros or 2.5 study participant points (applicable for psychology students). If participants dropped out, they received the proportional amount of money and/or points. Additionally, participants were offered the opportunity to see their individual data after the study had been completed These individual data reports were provided as an RMarkdown file and participants were invited to discuss those with the research assistant, which gave important additional insights into their data (see Olthof et al. (2024)). More information about the project can be found at https://osf.io/fx3ay/?view_only=6f02cfa5e3784a87973b760d49d70776.

Average compliance per participant (i.e., number of completed evening questionnaires) was 37.76 (*SD =* 13.07 ; range = 7 - 60), which is equal to an average of 63.84% completed assessments (*SD =* 19.39%; range = 16.39% - 98.36%). Out of a total of 4,630 sent beeps, 2983 (64.43%) notifications were completed across participants. In line with other studies (e.g., Fuller-Tyszkiewicz et al., 2013; Ono et al., 2019; Rintala et al., 2019), compliance declined across the 60 days of data collection, where 84.32% of all notifications were answered in the first data collection week and 48.83% in the last data collection week. Individual compliance was not associated with any of the demographic (i.e., age, gender, being a student) and study variables (i.e., person-level aggregates of emotion characteristics and emotion regulation strategy use; all *p*’s ranging from .10 - .92) with one exception: Individuals with higher compliance had higher person-level aggregated scores on suppression (*r* = .31, *p* = .006).

### Measures

**Fig. 1:**
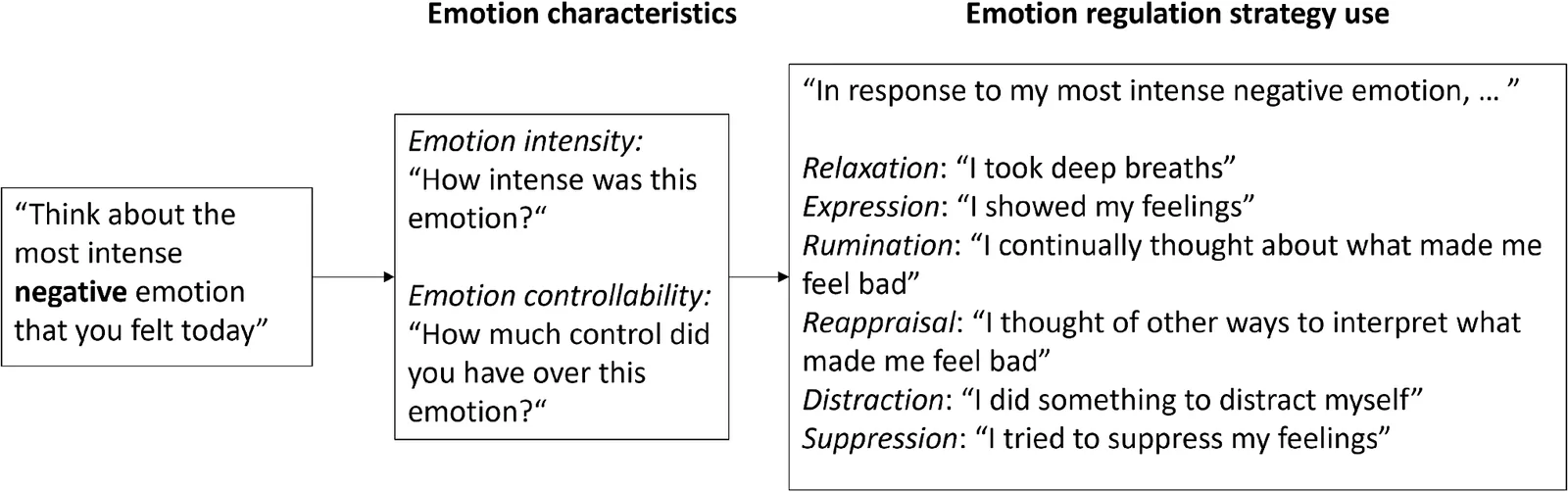
Questions on emotion characteristics and emotion regulation strategy use

To assess emotion characteristics and regulation strategy use, participants were asked at the end of each day to recall the most intense negative emotions and to describe that emotion with an open text box (not part of the present study). This was followed by questions about emotion characteristics and emotion regulation strategies (see Fig. 1).^2^

The items to assess emotion characteristics were based on items from Medland et al. (2020). Participants rated emotion intensity and controllability separately with a VAS response scale ranging from 0 (*not at all*) to 100 (*very intense*/*complete control*). The items to assess emotion regulation strategy use were based on the Regulating Emotion Systems Scale-Ecological Momentary Assessment (RESS-EMA) questionnaire (Medland et al., 2020). The RESS was originally designed as a trait questionnaire, but was adapted to the ESM context (Medland et al., 2020). It captures three different emotional response components, namely cognitive, behavioral and physiological (De France & Hollenstein, 2017). The cognitive component was measured with three emotion regulation strategies, namely distraction (i.e., diverting attention), rumination (i.e., sustained attention) and reappraisal (i.e., cognitive reframing). The behavior component was measured with two emotion regulation strategies, namely expression (i.e., active expression of/engagement in emotion) and suppression (i.e., inhibition of emotional expression). The physiological component was measured with one emotion regulation strategy, namely relaxation (i.e., dampening of autonomic arousal).

The original questionnaire has two items per subscale. For the present study, we selected one item per subscale using the following procedure: Using data from the original validation study (Medland et al., 2020), we calculated multilevel correlations and chose items with the strongest association between that item and momentary emotion intensity. We prioritized items that could be worded equivalently for both positive and negative emotions, because we used two items for positive emotion regulation (see Supplementary Material 1 for the results on positive emotion regulation). More information on this procedure is detailed at https://osf.io/fx3ay/files/osfstorage/62344ce61372e808bfcc59c2. All items were rated on a VAS response scale ranging from 0 (*not at all*) to 100 (*very much*). The RESS has good psychometric properties (De France & Hollenstein, 2017, 2019) and has been validated for the use in ESM studies (RESS-EMA; Medland et al. (2020)).

### Data Analysis

All analyses were conducted in *R* (R Core Team, 2023). We used the package *worcs* for the workflow (Version 0.1.14; Van Lissa et al., 2023] and the package *papaja* (Version 0.1.2; Aust and Barth, 2023) for manuscript preparation. For data preparation, we used *dplyr* (Version 1.1.3; Wickham, François et al. (2023)), *plyr* (Version 1.8.9; Wickham (2011)), *tidyr *(Version 1.3.0; Wickham, Vaughan et al. (2023)), and *esmpack* (Version 0.1.21; Viechtbauer and Constantin (2023)). Strategy switching was operationalized as the replacement subcomponent of the Bray-Curtis dissimilarity index and calculated using the package *betapart* (Version 1.6; Baselga et al., 2023). We used the package *misty* (Version 0.5.4; Yanagida (2023)) to calculate multilevel correlations and the *lme4* (Version 2.0.1, Bates et al., 2015) and *performance* (Version 0.17.0; Lüdecke et al., 2021) packages to calculate intraclass correlation (ICC) coefficients. Multilevel models were fit with the packages *nlme* (Version 3.1.162; Pinheiro and Bates (2000)) and *lmerTest* (Version 3.1.3; Kuznetsova et al. (2017)). Predicted values to evaluate the practical significance of quadratic effects were calculated with the emmeans package (Version 2.0.3, Lenth and Piaskowski, 2026).

#### Calculation of Strategy Switching Index

In addition to examining single emotion regulation strategies, we also examined the association between emotion characteristics and emotion regulation strategy switching. We quantified strategy switching as the replacement subcomponent of Bray-Curtis dissimilarity. Bray-Curtis dissimilarity is commonly used in ecology to quantify changes in biodiversity (Baselga, 2013). Recently, we showed that the replacement subcomponent detects strategy switching across measurement occasions when applied to emotion regulation ESM data (Lo et al., 2024). Moreover, this component is more sensitive in detecting dynamic emotion regulation variability than commonly used *SD*-based indices and consistently predicts decreases in negative affect (Lo et al., 2024), which since then has been replicated in independent samples (Zhu et al., 2026). A tutorial on how to calculate Bray-Curtis dissimilarity is publicly available on GitHub (Lo, 2023).

**Fig. 2:**
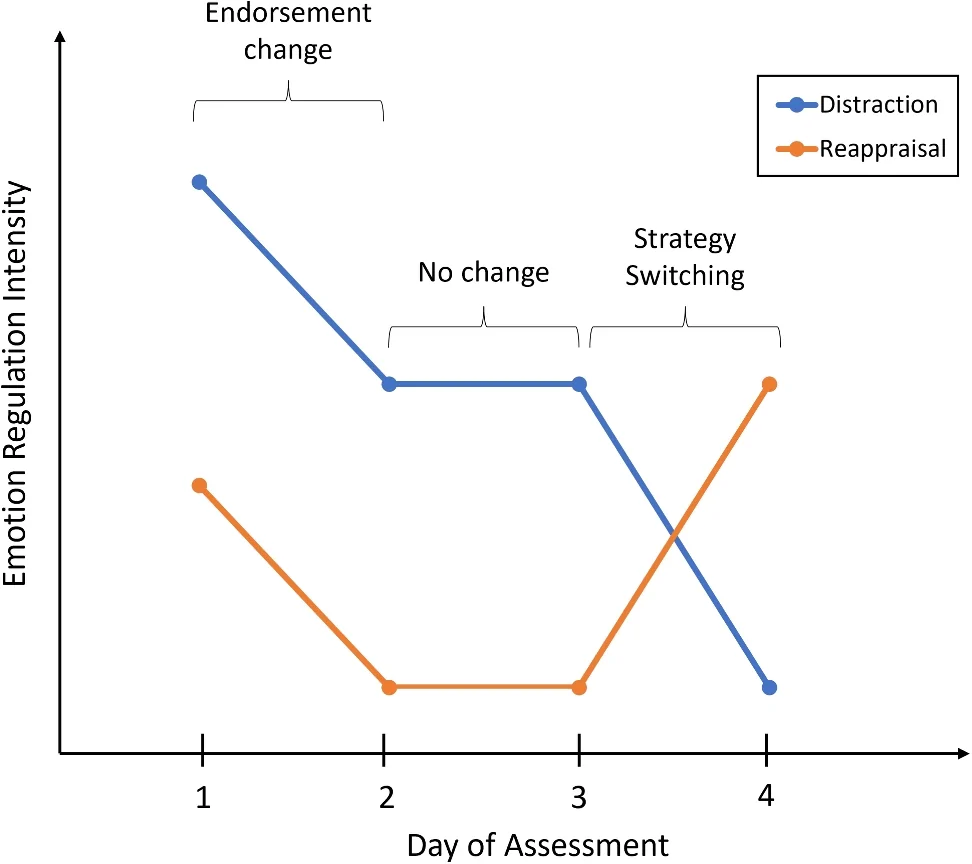
Subcomponents of emotion regulation variability: Endorsement change and strategy switching. Endorsement change refers to the overall change in endorsing emotion regulation strategies. Strategy switching refers to changing the composition of strategies. For the sake of clarity only two emotion regulation strategies are shown, but the index can handle more than two different strategies

The replacement subcomponent quantifies antagonistic changes between strategies (e.g., increases in one strategy, decreases in another strategy). It is calculated by comparing the moment of interest with all other moments the same adolescent reported, reflecting the within-person deviations from their typical strategy compositions (see also Lo et al., 2025). The index ranges from 0 to 1. A value of 0 indicates no switching in the strategy profile across days (i.e., all strategies change in the same direction across days, as when day 1 is compared against day 2 and day 3 in Fig. 2). A value of 1 indicates a complete switching from using one strategy to another across time points (e.g., a complete switch from distraction to reappraisal from day 3 to day 4 in Fig. 2).

Computing the replacement subcomponent (i.e., strategy switching) also yields the nestedness subcomponent, which captures overall change in effort across strategies (i.e., endorsement change), but not the direction of change, meaning that a significant coefficient could either refer to a collective increase or decrease in in the endorsement of strategies. In Fig. 2, this is depicted by the collective decreases in distraction and reappraisal from day 1 to day 2. Following a prior example that modelled Bray-Curtis dissimilarity as an outcome variable (Lo et al., 2025), we also estimated models with nestedness as the outcome. However, in the main text we focus on the replacement results (i.e., strategy switching), because overall change in effort is contributed by changes in effort of separate strategies, which are already covered in our multilevel models for individual strategies. We report the nestedness results (i.e., endorsement change) in Supplemental Materials 3.

#### Multilevel Models

Using the data from the evening questionnaires on daily emotion characteristics and emotion regulation strategy use (referring to the most intense emotional episode of that day), we examined the within-person association between emotion characteristics and emotion regulation strategy use with conducted multilevel regression analyses. We used the *nlme* package (Pinheiro & Bates, 2000) with the *optim* optimizer and a two-level structure: daily repeated measurements (level 1) nested within individuals (level 2). In those models, the emotion regulation variables (single strategies or emotion regulation variability) were the outcomes, and linear and quadratic terms of the emotion characteristics were the predictors.^3^ We included presence of micro-interventions (i.e., whether the participant received a prompt for a micro-intervention that day) and day of the study as time-varying covariates to control for effects of micro-interventions and time trends, and age and gender as time-invariant covariates. For all analyses, we included random intercepts and random slopes and their correlation for emotion characteristics (for the linear and quadratic terms). The errors were assumed to be Gaussian distributed and serially correlated. The serial correlation of the errors was modeled using an AR(1) process. We pre-registered to test for the significance of random effects to determine whether there was heterogeneity in the within-person effects. However, we excluded these analyses, because recent work highlights that variation is usually the norm, not the exception, and merely reporting heterogeneity without further addressing meaningful moderators has little added value (Vuorre et al., 2025). In addition, we compared the linear and quadratic model using a Loglikelihood Ratio test, to examine whether the quadratic model fit the data better beyond the mere significance of the fixed quadratic effect (not pre-registered).

Separate models were conducted for each model combination. In total, there were 16 models for the two different emotion characteristics (intensity and controllability) and the eight different emotion regulation outcomes (six single emotion regulation strategies and the daily strategy switching and endorsement change components). Remember that the endorsement change component was only reported in the supplement. Before analyses, emotion characteristics and emotion regulation strategy use (scale 0-100) were divided by 10 to prevent that the quadratic terms would become too large to avoid problems with model estimation. Moreover, to improve comparison with the analyses of the single emotion regulation strategies, we multiplied the strategy switching and endorsement change components of the Bray-Curtis dissimilarity index with 10, so that all variables fell on a scale from 0 to 10. Gender was recoded for analyses into female and other (male and non-binary). The time-varying predictors (i.e., micro-interventions, emotion characteristics) were person-mean centered and the time-invariant predictor (i.e., age) was grand-mean centered. Due to the large number of tests, we controlled for multiple testing, by adjusting our *p*-values for multiple comparisons using a Holm correction (Holm, 1979). Here, we included the *p*-values from all effects related to our research questions. 4

## Results

### Descriptive Statistics

**Fig. 3:**
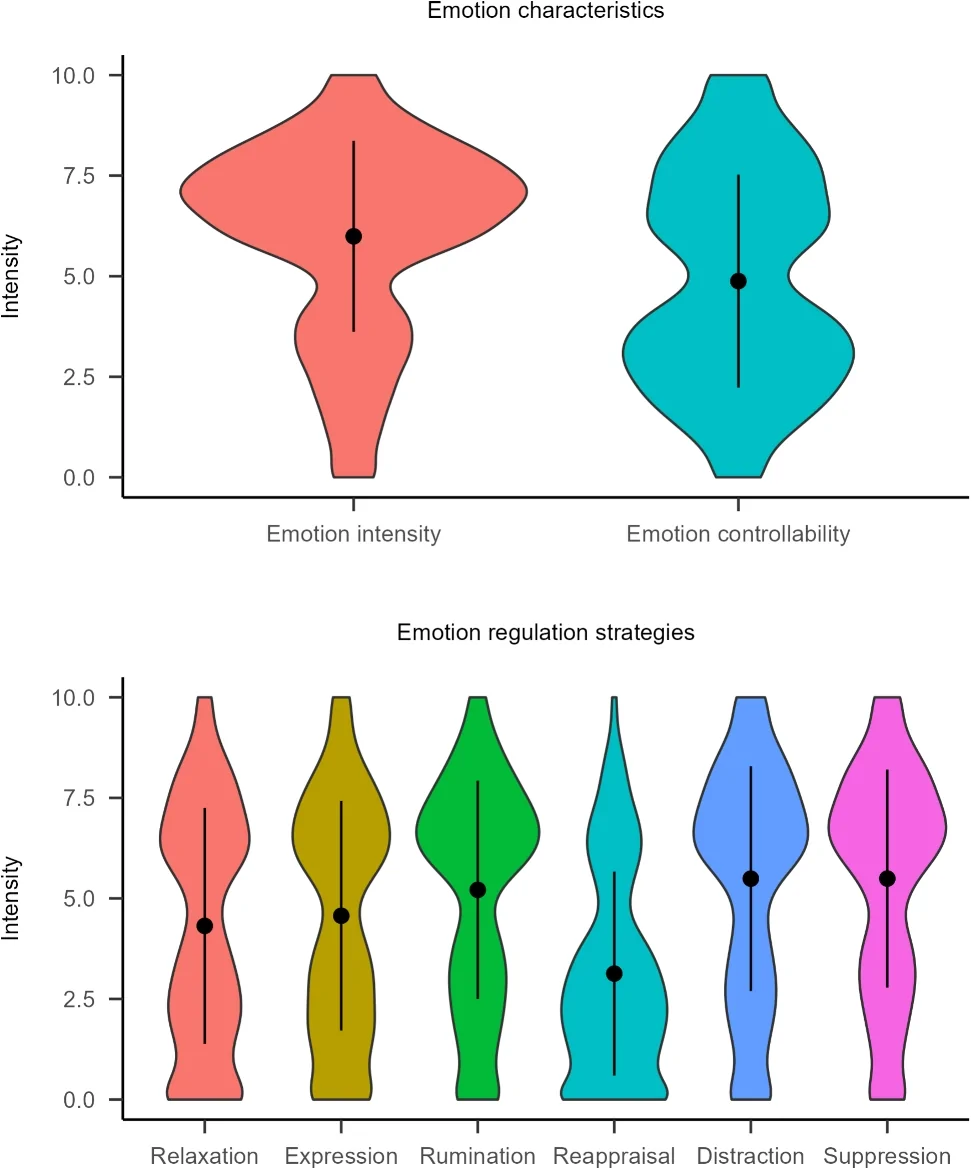
Violin plots of emotion characteristics and emotion regulation strategy use

Descriptive statistics can be found in Table 1 and the distribution of the variables are shown in Fig. 3. The emotion characteristics and emotion regulation strategy variables showed a large spread with few floor effects. Paired *t*-tests of person-aggregated mean scores across time indicate that distraction, suppression, and rumination were used most in daily life (which did not differ from each other, *t*’s = -1.67 − 0.38, all *p*’s > .10), followed by relaxation and expression (which did not differ from each other, *t* = -1.15, *p* = .25), whereas reappraisal was used least. The test-statistics of the significant comparisons ranged from *t* = 3.97 − 14.75, all *p*’s < .001. The ICC’s for emotion characteristics ranged between 0.13 and 0.31, indicating that 69 − 87% of the variance in emotion characteristics and emotion regulation strategies occurred at the within-person level.

**Table 1:** Descriptive statistics for the emotion characteristics and emotion regulation strategies

	Mean	Min	Max	*SD*	% 0 resp	ICC
Emotion intensity	6.02	2.87	8.41	1.10	1.94	.19
Emotion controllability	4.84	1.88	8.29	1.44	2.38	.28
Relaxation	4.33	0.46	8.52	1.67	12.30	.31
Expression	4.53	1.74	7.68	1.15	8.65	.13
Rumination	5.15	1.49	7.98	1.18	5.83	.16
Reappraisal	3.11	0.19	6.25	1.44	15.09	.31
Distraction	5.42	2.00	8.51	1.16	5.87	.14
Suppression	5.36	2.01	8.64	1.28	4.93	.19

*Note.**M* = Mean, *SD* = Standard Deviation, Min = Minimum, Max = Maximum, %0 resp = % of responses where participants endorsed a *0*, ICC = Intraclass Correlation. Mean, *SD*, minimum and maximum represent the descriptives of the person-aggregated scores for each emotion regulation strategy

Correlations at the between-person and within-person level are presented in Table 2. Emotion intensity and controllability were negatively related on a between-person and within-person level, indicating that individuals that reported higher negative emotion intensity than others, also reported lower controllability over their negative emotions (between-person level) and that on days when participants reported more intense negative emotions, they also reported lower controllability (within-person level). The different emotion regulation strategies were also generally positively correlated. On a between-person level, results indicated that individuals who on average used one emotion regulation strategy more than other individuals, also tended to use other emotion regulation strategies to a greater extent. This was the case for all strategies, except suppression which was not related to relaxation, expression and reappraisal on a between-person level. Similarly, the single emotion regulation strategies were overall positively related on a within-person level, indicating that on days where individuals used one emotion regulation strategy to a greater extent, they also used other emotion regulation strategies to a greater extent. The only exception was expression and suppression, for which a negative within-person correlation was found. This came as unsurprising, as it is difficult to both outwardly express and simultaneously inhibit emotional responses.

**Table 2:** Within-person and between-person correlation of emotion characteristics and emotion regulation strategies

	1.	2.	3.	4.	5.	6.	7.	8.
1. Negative emotion intensity		-.43***	.23***	.39***	.54***	.12***	.12***	.19***
2. Negative emotion control	-.42***		-.02	-.17***	-.33***	-.03	.02	-.08***
3. Relaxation	.25*	.17		.21***	.19***	.09***	.08***	.17***
4. Expression	.38**	.13	.44***		.30***	.06**	.04*	-.07***
5. Rumination	.67***	-.13	.45***	.29*		.13***	.09***	.21***
6. Reappraisal	.26*	.20	.60***	.58***	.50***		.14***	.15***
7. Distraction	.13	.35**	.33**	.43***	.30*	.50***		.26***
8. Suppression	.34**	-.14	.20	.01	.49***	.15	.45***	

*Note.* Above the diagonal represent the within-person correlations, below the diagonal between-person correlations, *p< .05, **p< .01, ***p< .001

### Multilevel Models

**Fig. 4:**
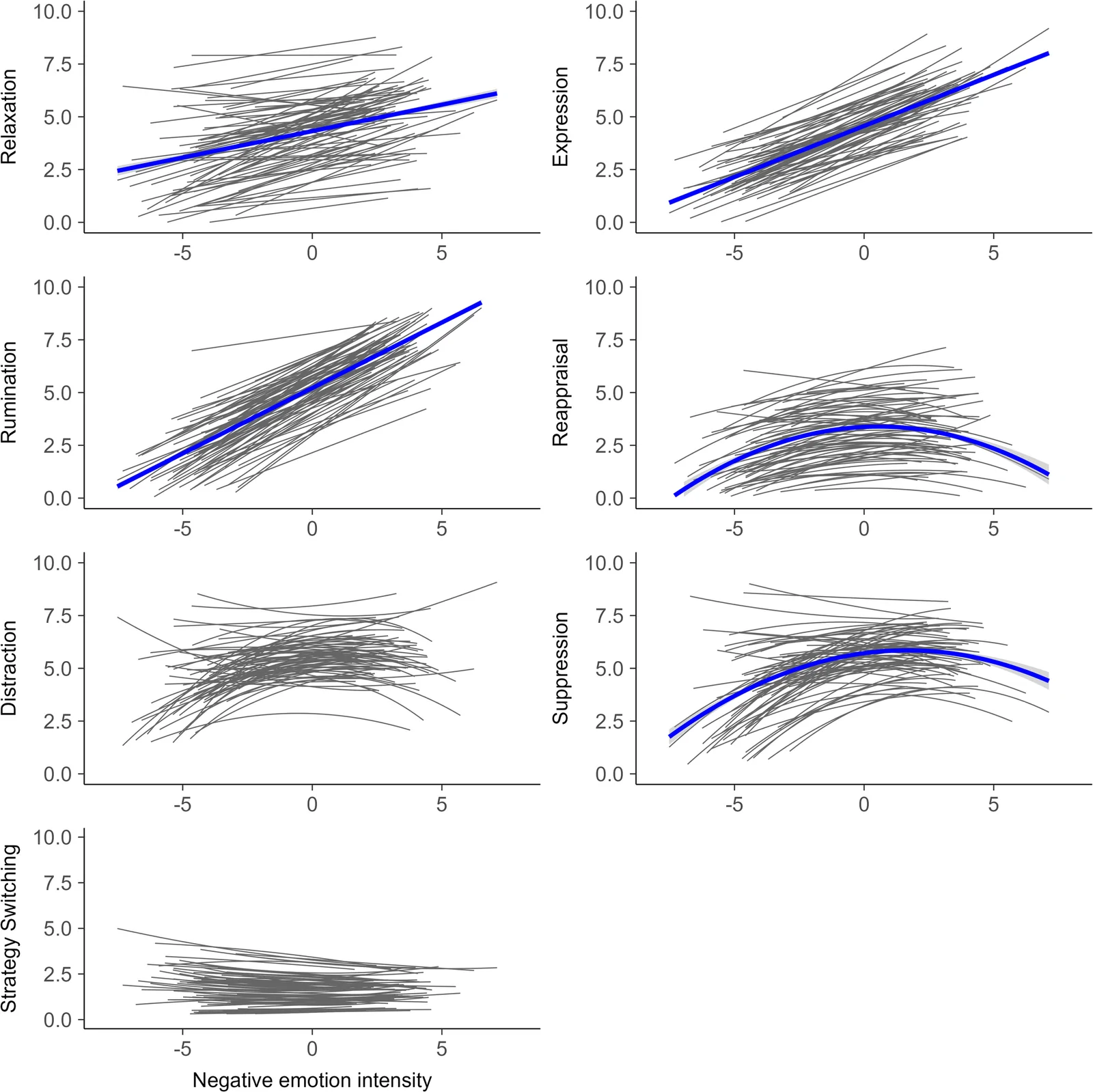
Within-person association between person-centered emotion intensity and emotion regulation strategy use. Blue, thick lines depict average fixed effects, grey, thin lines depict individual effects. Distraction and Strategy Switching showed neither linear nor quadratic associations with emotion intensity, thus we did not add the average fixed effect in the figure

The results of the multilevel models for emotion characteristics and emotion regulation strategies are presented in Table 3 and Figs. 4 (for intensity) and 5 (for controllability). We present quadratic model results because they in most cases fit the data better than - or similarly well as - the linear models (see Supplementary Material 4).^5^

For emotion intensity, we found positive linear, but no quadratic within-person effects for relaxation, expression, and rumination: On days when participants reported higher negative emotion intensity, they also reported greater use of relaxation, expression, and rumination. In contrast, we found quadratic effects for reappraisal and suppression, indicating that on days that individuals reported lower or higher than average negative emotion intensities, they also reported using less reappraisal and suppression. In other words, reappraisal and suppression were particularly used on days where participants reported negative emotions of average intensities (i.e., values close to an individual’s own average). For distraction, no significant linear and quadratic effects were found.

For emotion controllability, we found negative linear, but no quadratic effects for expression, indicating that on days that participants reported that their emotions were less controllable, they also reported to express them to a greater extent. Additionally, we found quadratic effects for rumination, reappraisal and suppression, indicating that on days where individuals reported very uncontrollable or very controllable emotions, they reported to use less reappraisal, rumination, and suppression (i.e., these strategies were particularly used with emotions of average controllability). No significant linear and quadratic effects were found for relaxation and distraction.

**Table 3:** Within-person association between emotion intensity and controllability with emotion regulation outcomes (Single Strategies and Emotion Regulation Variability)

	Intensity - Linear			Intensity - Quadratic			Control - Linear			Control - Quadratic		
ER outcomes	est	*SE*	$p_{adj}$	est	*SE*	$p_{adj}$	est	*SE*	$p_{adj}$	est	*SE*	$p_{adj}$
Relaxation	**0.24**	**0.04**	$\boldsymbol{< .001}$	-0.01	0.01	1.000	-0.04	0.03	1.000	-0.04	0.01	.072
Expression	**0.49**	**0.03**	$\boldsymbol{< .001}$	0.02	0.01	.963	**-0.20**	**0.03**	$\boldsymbol{< .001}$	-0.03	0.01	.145
Rumination	**0.62**	**0.03**	$\boldsymbol{< .001}$	0.01	0.01	1.000	**-0.36**	**0.04**	$\boldsymbol{< .001}$	**-0.06**	**0.01**	$\boldsymbol{< .001}$
Reappraisal	0.08	0.03	.186	**-0.03**	**0.01**	**.004**	-0.03	0.03	1.000	**-0.04**	**0.01**	$\boldsymbol{< .001}$
Distraction	0.09	0.03	.189	-0.03	0.01	.092	0.00	0.03	1.000	-0.03	0.01	.079
Suppression	**0.17**	**0.04**	$\boldsymbol{< .001}$	**-0.04**	**0.01**	$\boldsymbol{< .001}$	**-0.11**	**0.03**	**.024**	**-0.04**	**0.01**	**.007**
Strategy Switching	-0.02	0.01	.782	0.01	0.00	.550	0.00	0.01	1.000	**0.01**	**0.00**	**.003**

*Note.**p*-values are adjusted for multiple testing using a Holm correction (Holm, 1979). Significant fixed effects are printed in **bold**

**Fig. 5:**
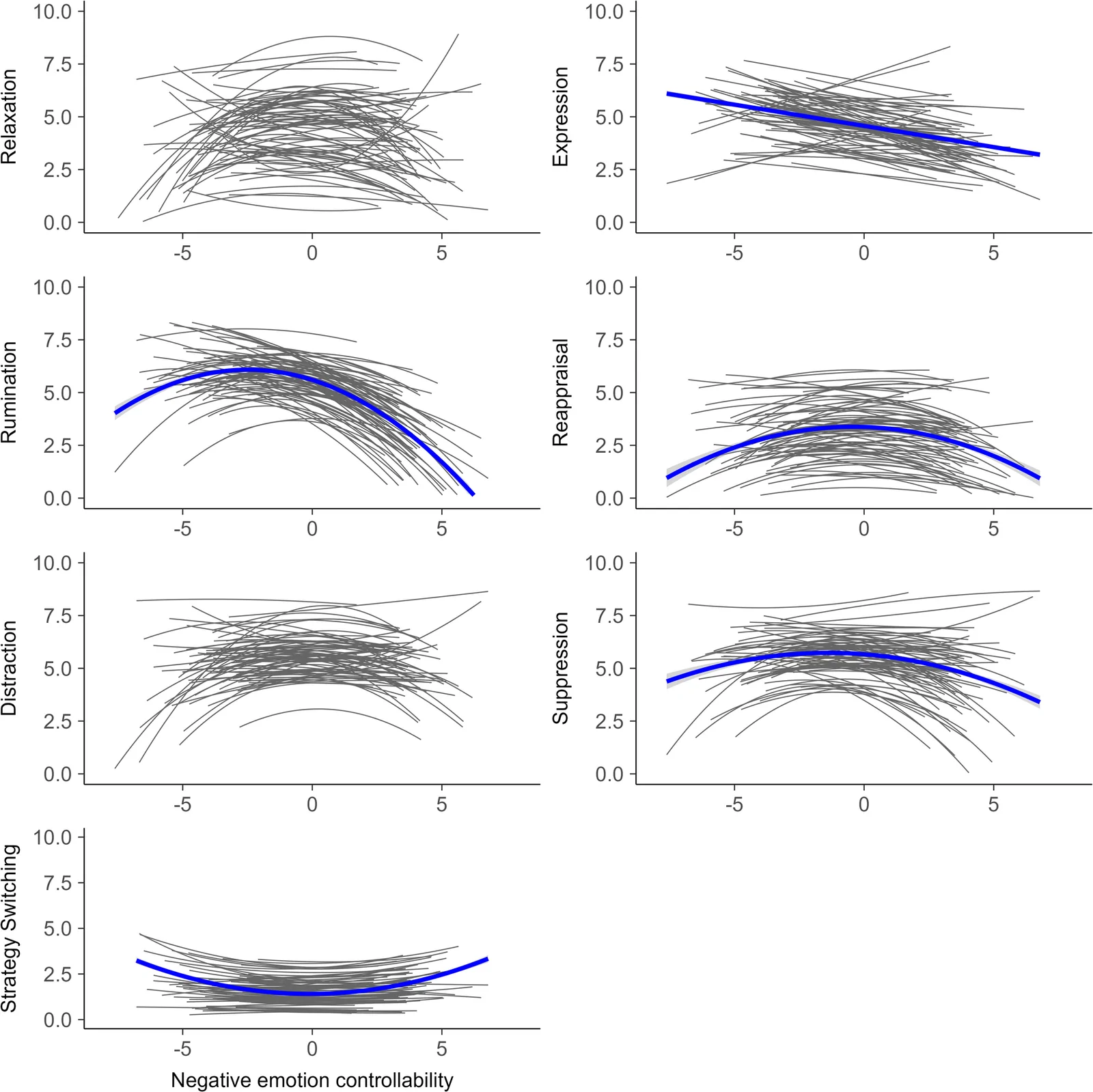
Within-person association between person-centered emotion controllability and emotion regulation strategy use. Blue, thick lines depict average fixed effects, grey, thin lines depict individual effects. Relaxation and distraction showed neither linear nor quadratic associations with emotion controllability, thus we did not add the average fixed effect in the figure

### Exploratory Analyses

For the first set of exploratory analyses, we examined the interaction between emotion intensity and emotion controllability on emotion regulation strategy use. None of the interaction terms were significant (all *p*’s > .220; see Supplementary Material 5). For the second set of analyses, we examined the effect of emotion intensity and controllability on emotion regulation strategy switching, as calculated with the replacement component of the Bray-Curtis dissimilarity index. Results are presented in Table 3 and Figs. 4 (for intensity) and 5 (for controllabilty). For emotion intensity, neither the linear, nor the quadratic effect was significant. For emotion controllability, we found linear and quadratic effects. On days that participants experienced emotions that were either more or less controllable than usual, participants engaged more in strategy switching.

To evaluate the practical significance of the quadratic effects, we plotted predicted values of emotion regulation strategy outcomes for different values of the emotion characteristic predictors. Here, we focused on models where the quadratic effects were significant after correction for multiple testing. The results are depicted in Table 4. Although several quadratic effects were statistically significant, predicted values indicated that most effects corresponded to relatively modest changes in emotion regulation strategy use (generally less than one point on a 0 to 10 scale).

**Table 4:** Predicted values of emotion regulation strategies at low, mean, and high levels of within-person predictors emotion intensity and controllability

Model (Predictor - Outcome)	Low (-2 *SD*)	Mean	High (+2 *SD*)
Intensity - Reappraisal	2.20	3.09	2.85
Intensity - Suppression	3.88	5.32	5.28
Controllability - Reappraisal	2.45	3.10	2.21
Controllability - Suppression	4.97	5.21	3.97
Controllability - Rumination	5.75	5.41	2.60
Controllability - Strategy Switching	1.82	1.37	2.16

*Note.* Covariates were held constant at their reference category (for categorical variables) or mean (for continuous variables). Assessment time was centered on the middle observation (i.e., day 30 of day 60). Predicted values are reported on the original outcome scale (0–10). Because predictions are based on both linear and quadratic terms, changes between -2 *SD* and +2 *SD* are not necessarily symmetric around the mean

## Discussion

This study examined how emotion characteristics are associated with emotion regulation strategy use in daily life and provided novel evidence for quadratic relations for some strategies. Specifically, individuals endorsed suppression and reappraisal particularly on days when their emotions were of average intensity and controllability, and less on days where emotions were either relatively low or high in those characteristics. Moreover, individuals tended to switch to strategies they do not typically use when emotion intensity and controllability were at their extremes. Our findings highlight the value of moving beyond linear relations when studying emotion regulation in daily life.

### Quadratic Relations Between Emotion Characteristics and Emotion Regulation

We replicated earlier linear findings (e.g., De France and Hollenstein, 2022; Medland et al., 2020), showing that individuals used more relaxation, expression and rumination with increasing emotion intensity and uncontrollability (for expression only). More importantly, we found several inverted U-shaped relations, most notably for suppression and reappraisal, which were used most with emotions of average intensity and controllability. Suppression is a disengagement strategy (Silk et al., 2003), and laboratory research has shown that individuals prefer suppression with intense stimuli (Sheppes, 2020). However, our results suggest that individuals may use suppression less if emotions become *too* intense or uncontrollable. These results make sense considering that suppression is an act of self-control (Geisler & Schröder-Abé, 2015), which may become too difficult at the extremes.

Reappraisal, the strategy used least overall, showed similar patterns. While laboratory studies often find that reappraisal is used with low intensity stimuli, our results suggest that in daily life individuals reappraise emotions of average intensity and controllability. At low intensities, reappraisal may not be deemed necessary. At high intensities, it may become too cognitively demanding (Sheppes, 2020), prompting a preference for less effortful strategies (e.g., expression). This discrepancy with laboratory studies may stem from the nature of low-intensity emotions in daily life. Examining narrative descriptions that our participants provided using open text-boxes revealed relative mundane situations and emotions (e.g., boredom during meetings, frustration during studying), which individuals may not be motivated to reappraise. These situations are quite different from the low-intensity stimuli typically seen in laboratory studies (e.g., mild electric shocks; Sheppes et al. (2011)). Apart from that, laboratory paradigms typically ask individuals to engage in either strategy, thus capturing emotion regulation choice as a binary decision (chosen vs. not chosen), whereas in our study, individuals reported on the extent to which they engaged in emotion regulation on a continuous scale, which may either reflect initiation (i.e., deciding whether to regulate), effort (i.e., deciding the extent to which they regulate), and/or maintenance of emotion regulation (i.e., deciding how long to maintain regulation; Tamir (2021)). For rumination, we found linear associations with intensity, but quadratic relations for controllability, suggesting that rumination decreased slightly on days that emotions were more uncontrollable than usual. Although this decrease was small and individuals ruminated least with controllable emotions, the results are puzzling because rumination is an involuntary engagement strategy (e.g., Silk et al., 2003), which should be expected with uncontrollable emotions. Contrary to laboratory studies showing increased distraction in intense situations (e.g., Matthews et al., 2021), we found, in line with other ESM studies (Blanke et al., 2022; De France & Hollenstein, 2022; Medland et al., 2020), no evidence for such an association. It is possible that individuals are not aware of the short-term benefits of distraction (Blanke et al., 2022). Another explanation is the relatively young age of our sample, because research suggests that older adults choose distraction more easily than younger adults in intense situations (Scheibe et al., 2015). Contrary to earlier research (Petrova et al., 2024), we found no interaction effects between intensity and controllability.

Taken together, our results suggest that relative demanding strategies, such as suppression and reappraisal, are less strongly associated with overwhelming emotions (i.e., high-intensity and/or uncontrollable emotions), whereas involuntary strategies, such as rumination and expression, are strongly associated with overwhelming emotions. Similarly, relaxation, a voluntary, directed strategy that targets the physiological component of emotions, seems more likely with relatively intense emotions. The quadratic findings are also consistent with emerging evidence that individuals do not always attempt to regulate their emotions. Recent studies suggest that individuals may choose not to regulate when emotions are perceived as appropriate or insufficiently intense, and in some cases when emotions are very intense, but individuals do not know how to regulate them or feel incapable to (e.g., Daniel et al., 2024; Lai et al., 2026; Uchida et al., 2026). This perspective aligns with the observed quadratic patterns, suggesting that regulation efforts – at least for reappraisal and suppression – are most likely for average levels of emotion intensity and controllability.

Beyond *single* strategies, our results suggest that emotion characteristics were also associated with variability across *multiple* strategies, although only for controllability and not for intensity. The null results between intensity and switching were surprising, considering recent findings where greater switching was associated with decreases in negative emotion intensity (Lo et al., 2024). Switching may be more relevant for emotion regulation success and less relevant for the selection and maintenance of different regulation strategies.

In contrast, emotion controllability was quadratically related to strategy switching: Individuals switched to using typically less-used strategies on days were their emotions were more controllable or uncontrollable than usual. The rationale may differ at both ends of the curve: Highly controllable emotions could be safe situations for experimenting with unfamiliar strategies, while turning to rarely used strategies for highly uncontrollable emotions might be a last resort when usual strategies fail. Since the Bray-Curtis index only captures overall deviations in composition (i.e., it tells something about that strategies changes in relation to each other, but not between which strategies individuals alternated), future research could use mixture models to further explore qualitative differences in strategy composition (Grommisch et al., 2020).

### Limitations and Conclusion

This study has several limitations. First, some model combinations were underpowered, so results should be interpreted cautiously. Second, although university is a turbulent time requiring emotion regulation skills, our sample had predominantly educated, young, female university students, limiting generalizability.

Third, we asked participants to report about the most intense emotion and related regulation once per day. While this may decrease floor effects often found in ESM studies (e.g., Lai et al., 2026), it may have underrepresented lower-intensity experiences. Nevertheless, our data still showed substantial variability in both emotion characteristics and regulation strategies, suggesting that the design captured a meaningful range of emotional experiences. Moreover, while this instruction likely improved interpretability for participants, it increases ambiguity in interpreting the results, since end-of-day reports may still suffer from recall bias and because it is unclear whether the reported strategies reflect regulation at the peak of an emotional episode, averaged across the whole episode or global daily regulation. Therefore, our results, particularly regarding the results on strategy switching, could reflect both episode-specific and broader day-level processes. To better disentangle these processes, future studies could include event-triggered questionnaires, where participants answer questions about emotion regulation during salient moments (Hoemann et al., 2020; Revol et al., 2025).

Fourth, emotion characteristics and regulation strategies were assessed simultaneously. While we asked participants to *first* think about their most intense emotion, *then* to rate the intensity and controllability of their most intense emotion, and *then* their strategy use in response to that emotion, the emotion characteristics likely contain *antecedents* and *effects* of regulation. This is particularly the case for emotion controllability, which itself may be influenced by regulation success (i.e., an emotion that was successfully regulated may be perceived as more controllable in hindsight). Therefore, emotion characteristics should not be interpreted as purely antecedent variables. However, although process models emphasize distinguishing emotion generation and regulation (e.g., Gross, 2015), we agree with other researchers that these processes are inseparable, because they are continuously ongoing through positive and negative feedback loops (Hollenstein, 2015; Kappas, 2011).

Finally, using a limited set of predefined items may fail to capture the idiosyncrasy of emotion regulation. For instance, distraction could mean mindless phone scrolling or creating beautiful art - likely qualitatively different experiences. Emerging research where participants provide qualitative descriptions of their experiences (e.g., Bringmann et al., 2026; Hoemann et al., 2023) provide an exciting avenue to capture the diversity of emotion regulation. Despite these limitations, our study highlights the benefit of moving beyond linear relations when studying emotion regulation dynamics in daily life - both for separate strategies and multi-strategy switching.

## Supplementary Information

Below is the link to the electronic supplementary material.

Supplementary file 1 (pdf 46 KB)

## Footnotes

1 Our pre-registration erroneously stated that we would only examine the association between intensity and regulation variability, although we also planned to examine controllability. We additionally asked participants about positive emotions and two related strategies (savoring, positive expression). We pre-registered to test these relations as well, but report results in Supplementary Material 1 due to word constraints.

2 Note that before asking participants about their most intense negative *emotions*, we asked them about the most intense negative *event* they experienced, including content, intensity and impact. While some studies study the link between such *situational* characteristics and emotion regulation strategies, we decided to focus on *emotion* characteristics, because emotion regulation is about the regulation of an emotion, not a situation. In fact, when we asked participants whether their most intense emotion was related to the most intense event they just reported on, in 31% of the cases, the most intense negative emotion was *not* related to the most intense negative event.

3 An anonymous reviewer suggested adding analyses on how the type of emotions influenced effects (e.g., whether effects differed depending on whether anger or sadness was regulated). While we had information about which specific emotions participants regulated through open-ended responses, reviewing this information showed that it was not suitable to include in multilevel analyses. This is because participants sometimes reported on more than one emotion (e.g., “anxiety, stress, loneliness”, “sad and angry and irritated”) or on emotions that are not typically considered emotions (e.g., “apathy”, “dissociative”, “defeat”). Categorizing these data would also mean including many different categories in multilevel analyses, which would likely result in too little power. Thus, we did not include these extra analyses. Future research could explicitly add different emotion categories when assessing intensity and controllability to study how the association between emotion characteristics and emotion regulation strategies varies across emotions.

4 There were 38 effects in total: The *p*-values of the fixed linear and quadratic effects (2 effects [linear & quadratic] * 8 emotion regulation outcomes [six single emotion regulation strategies and the daily strategy switching and endorsement change components] * 2 emotion characteristics [intensity & controllability] = 32 effects) and the interaction effects between intensity and controllability for predicting six emotion regulation strategies of the exploratory analyses (6 effects).

5 Note that even if the model fit comparison indicates that the quadratic models fit be data better, it is still possible that the fixed quadratic effect is not significant. This isbecause a better fitting model may also be due to significant quadratic slope variances and/or significant correlations of the quadratic slope with the other model parameters.

## Additional Information

### Data Availability

The data can be downloaded at the university’s data repository after signing a Data Use Agreement (https://doi.org/10.34973/s90j-0r08).

### Code availability

The analysis code is available at https://osf.io/f42un/?view_only=ba769f827a04424389311f66db8fdeb1.
