# Supplementary material for: Too Little, Too Much: Evidence for Quadratic Patterns in Emotion Regulation Strategy Use in Daily Life
Source: Affect Sci. 2026 Jul 20;7(3):498–512. doi: 10.1007/s42761-026-00390-9 (PMC13582785; doi:10.1007/s42761-026-00390-9)
Supplement: Supplementary file 1 [file 42761_2026_390_MOESM1_ESM.pdf]

Supplementary Material - Too Little, Too Much: Evidence for Quadratic Patterns in Emotion Regulation Strategy Use in Daily Life

Dominique F. Maciejewski, Yasemin Erbas, Tak Tsun Lo, Egon Dejonckheere, Merlijn Olthof, Andrea Bunge, and & Eeske van Roekel

Author Note

Correspondence concerning this article should be addressed to Dominique F. Maciejewski, Warandelaan 2, 5037 AB Tilburg, the Netherlands. E-mail: d.f.maciejewski@tilburguniversity.edu

Supplementary Material - Too Little, Too Much: Evidence for Quadratic Patterns in Emotion Regulation Strategy Use in Daily Life

Supplementary Material

Supplementary Material 1. Results on positive emotion intensity and emotion regulation strategy use

In addition to asking about the most negative emotion and their associated emotion regulation strategy use, we also asked participants to report about their most positive emotions during that day and whether they employed two regulation strategy in response to their positive emotions. For positive emotions, we only assessed positive emotion intensity (and not controllability), because when we designed the study, we did not believe that emotion controllability would a major role for positive emotions. Because at the time of the study, there were no questionnaires available asking about positive emotion regulation, we adapted items from the RESS-EMA (Medland et al., 2020) we thought were most relevant to the maintenance of positive emotions, namely savoring (which is comparable to positive rumination; "I continually thought about what made me feel good") and expression ("I showed my feelings"). Participants were asked to indicate the intensity to which they used a certain emotion regulation strategy after being prompted with "In response to my most intense positive emotions,…". We pre-registered that positive emotion intensity would be related to increased use of savoring and emotion expression and explored both linear and quadratic associations. The p-values were by adjusted for multiple comparisons using a Holm correction (Holm, 1979).

For positive emotion intensity, we found only linear, but no quadratic effects for both positive expression (linear: est = 0.77, $SE = 0.04$, $p = < .001$; quadratic: est = 0.03, $SE = 0.01$, $p = .05$ and savoring (linear: est = 0.75, $SE = 0.03$, $p < .001$; quadratic: est = 0.00, $SE = 0.01$, $p = .77$), indicating that when participants reported more intense positive emotions, they reported a higher expression of their positive emotions and greater savoring. These results are in line with previous studies showing that positive emotions are related to more savoring and expression in daily life (Hiekkaranta et al., 2021; Vanderlind et al.,

2022).

Supplementary Material 2. Results Power Analyses

We conducted an a-priori power analysis using Monte Carlo Simulations with 1000 replications following procedures from Lafit et al. (2021) using parameter estimates from another pre-existing dataset (Medland et al., 2020). Results are shown in Supplementary Table 1. Notably, while quadratic effects typically require larger samples, which may explain why power tended to be larger for quadratic compared to linear terms, our power analyses showed that the power to detect quadratic effects was sometimes higher than for linear effects. This may be partly explained by our approach, because the power analyses were based on estimates from a prior study. This reflects that, in the presence of a true U-shaped relation in the study of Medland et al. (2020), the linear component can be close to zero because increases and decreases across the range cancel each other out, resulting in low power to detect this small effect. In contrast, the quadratic term captures the curvature of the relationship and may therefore have a larger effect size, leading to higher power. For code and more information see our pre-registration https://osf.io/fg58t/?view_only=7711b0c0057346f58d10192a324ae817.

Table 1

Results Power Analyses

	Intensity		Control	
Predictor	linear effect	quadratic effect	linear effect	quadratic effect
relaxation	1.00	0.18	0.21	0.95
expression	0.97	0.09	0.19	0.18
rumination	1.00	0.18	0.21	0.62
reappraisal	0.95	0.32	0.71	0.82
distraction	0.75	0.29	0.26	0.56
suppression	1.00	0.45	0.06	0.93

Supplementary Material 3. Results on Endorsement Change supcomponent Bray-Curtis dissimilarity index

The Bray-Curtis dissimilarity index has two subcomponents: Replacement, which captures compensatory changes between strategies (i.e., strategy switching), and nestedness, which captures overall change in effort across strategies, but not the direction of change. In the main text, we focus on the replacement results, because overall change in effort is contributed by changes in effort of separate strategies, which are already covered in our multilevel models for individual strategies. Here, we report the results on the nestedness component (see Supplementary Table 2).

For emotion intensity, we found linear and quadratic effects (i.e., the deviation in daily intensity in using emotion regulation strategies compared to the person-mean). This indicates that on days that individuals reported lower or higher negative emotion intensity than average, they deviated more from their typical emotion regulation effort. For emotion controllability, we found significant quadratic effects, indicating that when emotions were

either less controllable or more controllable than usual, participants deviated more from their usual emotion regulation style in the overall effort of using strategies.

Because this component only reflects deviations from average strategy use, but not their direction, it is unclear whether individuals initiated or inhibited emotion regulation efforts with low versus high intensity and controllable emotions. Results on the single strategies give more insight into that. For instance, individuals used less relaxation, expression and rumination with few intense emotions and more relaxation, expression and rumination with more intense emotions. In contrast, individuals showed less reappraisal and suppression with both low and high intense emotions.

Table 2

Within-person associations of negative emotion intensity and controllability with endorsement change

	Linear			Quadratic		
ER outcomes	est	SE	p_{adj}	est	SE	p_{adj}
Negative emotion intensity	**-0.08**	**0.01**	**$<$ .001**	**0.04**	**0.00**	**$<$ .001**
Negative emotion controllability	0.04	0.01	.090	**0.03**	**0.01**	**$<$ .001**

Note. p-values are adjusted for multiple testing using a Holm correction (Holm, 1979). Significant fixed effects are printed in **bold**.

Supplementary Material 4. Model comparisons for linear versus quadratic models

The results on model comparisons along with fit indices are presented in Supplementary Table 3 (for intensity) and 4 (for controllability). While for emotion controllability the AIC, BIC and Loglikelihood Ratio test all favored the quadratic model, for emotion intensity, the BIC only favored the quadratic model for distraction, suppression, and endorsement change (whereas the AIC and Loglikelihood ratio test additionally favored the quadratic model for relaxation, reappraisal and strategy switching). Note that instances where the BIC and AIC as well as the Loglikelihood ratio test disagreed concerned models where the quadratic effect was not significant (except for reappraisal).

Table 3

Model fit results comparing linear with quadratic models for emotion intensity

ER strategies	AIC linear	AIC quadratic	Δ AIC	BIC linear	BIC quadratic	Δ BIC	Likelihood ratio	*p*
Relaxation	13648.10	13644.46	3.64	13714.11	13734.47	-20.36	11.64	.020
Expression	13990.65	13992.01	-1.36	14056.66	14082.02	-25.36	6.64	.156
Rumination	12981.55	12985.29	-3.74	13047.56	13075.30	-27.74	4.26	.372
Reappraisal	13018.46	13005.51	12.95	13084.47	13095.52	-11.05	20.95	$< .001$
Distraction	14238.08	14211.31	26.77	14304.09	14301.32	2.77	34.77	$< .001$
Suppression	13722.85	13691.73	31.12	13788.86	13781.74	7.12	39.12	$< .001$
Strategy Switching	5769.33	5756.32	13.01	5835.23	5846.18	-10.95	21.01	$< .001$
Endorsement Change	6942.40	6610.19	332.21	7008.30	6700.06	308.25	340.21	$< .001$

Note. For the model comparison, the models were refitted with Maximum Likelihood estimation because REML (Restricted Maximum Likelihood) is not appropriate when the models have different fixed effects.

Table 4

Model fit results comparing linear with quadratic models for emotion controllability

ER strategies	AIC linear	AIC quadratic	Δ AIC	BIC linear	BIC quadratic	Δ BIC	Likelihood ratio	*p*
Relaxation	13782.31	13700.38	81.93	13848.32	13790.39	57.93	89.93	< .001
Expression	14338.95	14297.14	41.81	14404.95	14387.15	17.80	49.81	< .001
Rumination	13612.95	13535.80	77.15	13678.96	13625.81	53.15	85.15	< .001
Reappraisal	13054.55	13025.61	28.94	13120.55	13115.62	4.94	36.94	< .001
Distraction	14272.59	14236.13	36.46	14338.60	14326.14	12.46	44.46	< .001
Suppression	13866.38	13823.33	43.05	13932.39	13913.34	19.05	51.05	< .001
Strategy Switching	5808.31	5723.04	85.27	5874.21	5812.91	61.31	93.27	< .001
Endorsement Change	7216.14	7089.40	126.74	7282.04	7179.27	102.78	134.74	< .001

Note. For the model comparison, the models were refitted with Maximum Likelihood estimation because REML (Restricted Maximum Likelihood) is not appropriate when the models have different fixed effects.

Supplementary Material 5. Results on Interaction Effects

Supplementary Table 5 shows the model coefficients for the interaction effects between emotion intensity and controllability for all six emotion regulation strategies.

Table 5

Interaction Effect of Emotion Intensity and Emotion Controllability on Emotion Regulation Strategies

ER outcomes	est	SE	p_{adj}
Relaxation	0.00	0.01	1.000
Expression	-0.02	0.01	1.000
Rumination	-0.02	0.01	.220
Reappraisal	0.02	0.01	.416
Distraction	0.01	0.01	1.000
Suppression	0.01	0.01	1.000

Note. ER = Emotion regulation. p-values are adjusted for multiple testing using a Holm correction (Holm, 1979)

References

Hiekkaranta, A. P., Kirtley, O. J., Lafit, G., Decoster, J., Derom, C., de Hert, M., Gülöksüz, S., Jacobs, N., Menne-Lothmann, C., Rutten, B. P. F., Thiery, E., van Os, J., van Winkel, R., Wichers, M., & Myin-Germeys, I. (2021). Emotion regulation in response to daily negative and positive events in youth: The role of event intensity and psychopathology. *Behaviour Research and Therapy*, *7001*, 103916–103916. https://doi.org/10.1016/j.brat.2021.103916

Holm, S. (1979). A simple sequentially rejective multiple test procedure. *Scandinavian Journal of Statistics*, *6*(2), 65–70. https://www.jstor.org/stable/4615733

Lafit, G., Adolf, J. K., Dejonckheere, E., Myin-Germeys, I., Viechtbauer, W., & Ceulemans, E. (2021). Selection of the number of participants in intensive longitudinal studies: A user-friendly shiny app and tutorial for performing power analysis in multilevel regression models that account for temporal dependencies. *Advances in Methods and Practices in Psychological Science*, *4*(1), 2515245920978738. https://doi.org/10.1177/2515245920978738

Medland, H., De France, K., Hollenstein, T., Mussoff, D., & Koval, P. (2020). Regulating emotion systems in everyday life. *European Journal of Psychological Assessment*, *36*(3), 437–446. https://doi.org/10.1027/1015-5759/a000595

Vanderlind, W. M., Everaert, J., & Joormann, J. (2022). Positive emotion in daily life: Emotion regulation and depression. *Emotion*, *22*(7), 1614–1624. https://doi.org/10.1037/emo0000944
